# Clinical Model Autophagy: The Risk of Interpretative Drift in Recursive Medical AI

**DOI:** 10.2196/94813

**Published:** 2026-04-21

**Authors:** Pei Fan Shih

**Keywords:** large language models, electronic health records, model collapse, data governance, diagnostic errors

## Abstract

The rapid integration of large language models into electronic medical record systems introduces a critical theoretical vulnerability. Drawing on foundational computer science proofs of “model collapse,” this viewpoint introduces the concept of “Clinical Model Autophagy”—a systemic degradation of diagnostic integrity that occurs when clinical artificial intelligence (AI) models are recursively trained on unverified, AI-generated synthetic data. As these recursive models may progressively regress toward statistical means, they undergo “Interpretative Drift,” a clinically concerning phenomenon where rare pathological variances are systematically erased and complex diseases are homogenized into benign averages. To prevent the irreversible contamination of health care data ecosystems, the author urgently proposes the Data Purity Standard (DPS). The DPS mandates the cryptographic watermarking of all AI-assisted clinical entries for provenance tracking, alongside the establishment of “Human Vaults.” These physically segregated repositories of physician-verified heritage data will serve as immutable biological anchors to safely guide future AI training, ensuring the long-term reliability of digital health infrastructure.

## The Impending Threat of Recursive Artificial Intelligence Training

The integration of large language models (LLMs) into health care systems to draft clinical notes, summarize patient encounters, and synthesize documentation is accelerating at an unprecedented pace. While these tools offer undeniable administrative efficiencies, their unchecked deployment introduces a profound theoretical vulnerability to the medical data ecosystem [1]. In computer science, recent foundational proofs have definitively established that training generative artificial intelligence (AI) on synthetic, AI-generated data inevitably leads to an irreversible statistical degradation known as “model collapse” [2]. As recursive loops of generated content re-enter the training pipeline, models progressively forget the true underlying data distribution. Extrapolating this machine learning phenomenon to closed-loop electronic medical record (EMR) systems, the author posits that such a self-referential cycle poses a severe theoretical risk of triggering a phenomenon defined here as Clinical Model Autophagy—a systemic, self-consuming degradation of diagnostic integrity [1]. In this viewpoint, Clinical Model Autophagy is introduced as a theoretical risk framework intended to describe how recursive synthetic exposure could degrade medical AI systems under plausible closed-loop deployment conditions.

## Defining the Terminology: Endogenous vs Exogenous Risks

To ensure conceptual clarity, it is necessary to formally define these newly proposed terms and distinguish them from established terminology. In computer science, model collapse refers to the general statistical degradation observed when any generative AI trains on synthetic data. The author proposes the term Clinical Model Autophagy to describe the application-specific manifestation of this phenomenon within closed-loop EMR systems, highlighting the self-consuming erosion of clinical diagnostic integrity. Furthermore, Clinical Model Autophagy drives Interpretative Drift. This novel concept must be sharply distinguished from traditional concept drift or dataset shift, which occurs when the external, real-world data distribution changes over time (eg, emerging epidemiological trends). In contrast, Interpretative Drift is an endogenous, self-inflicted smoothing of data variance where the model actively erases rare pathologies despite the external real-world prevalence remaining constant. Finally, while automation bias describes a clinician's well-documented psychological tendency to over-rely on automated systems, Interpretative Drift describes the specific machine-side pathogenic mechanism that dangerously exploits this human vulnerability by generating highly fluent, yet clinically homogenized outputs.

## The Statistical Mechanics of Interpretative Drift

The mechanism driving this impending collapse is fundamentally rooted in the statistical architecture of LLMs, which operate by optimizing for linguistic fluency and probabilistic likelihood [3]. In human populations, diagnostic value is inherently concentrated at the long tails of clinical distributions, encompassing rare diseases, atypical presentations, and complex, multi-system pathophysiologies [1]. However, as AI-generated EMR data permeate hospital databases and are subsequently harvested to train future iterations of clinical models, a mathematical regression to the mean is triggered. The recursive ingestion of synthetic clinical text systematically erodes these pathological tail-end variances [4]. Because such models favor the most frequent and statistically expected tokens, recursive synthetic exposure may increasingly overwrite nuanced or severe clinical phenomena with homogenized language [1]. Ultimately, this homogenization forces the system's representation of human physiology to regress toward generic statistical averages, actively erasing the diversity of disease and defaulting to “healthy” or “normal” archetypes [1,4].

## The Clinical Cascade: Interpretative Drift in Action

The clinical consequence of this regression is a highly insidious threat this article terms as Interpretative Drift. Unlike early-generation AI failures that manifested as obvious syntactic gibberish or easily detectable language breakdown, Interpretative Drift produces outputs that maintain perfect structural and grammatical fidelity. To explain this conceptual framework, consider an illustrative thought experiment involving an oncology positron emission tomography/computed tomography report. While this specific clinical cascade remains speculative and has not yet been empirically observed at scale, it highlights the potential trajectory of interpretation errors. A future LLM, suffering from Clinical Model Autophagy, processes the imaging data and successfully extracts the correct numerical values, accurately documenting a focal lesion with an SUVmax of 12.4. However, due to Interpretative Drift, the model confidently hallucinates a benign clinical context, describing this hypermetabolic activity as normal “physiological uptake” rather than correctly identifying it as “progressive malignancy.” This subtle failure represents an insidious shift in diagnostic error paradigms. By coupling accurate numerical extraction with a hallucinatory but statistically smoothed interpretation, the model may superficially mimic professional competence, thereby attenuating the cognitive cues that would normally alert clinicians to diagnostic discrepancies [1,5]. In time-pressured clinical environments, such false reassurance may bypass casual human review, effectively transforming high-risk pathologies into benign administrative records and creating substantial patient-safety risk that may not be readily detected during routine review.

## A Precautionary Governance Framework: The Data Purity Standard

To prevent the irreversible contamination of our digital health infrastructure, the medical informatics community must transition immediately from passive monitoring to structural regulation. Historically, data governance in health care has been guided by established paradigms such as the FAIR (Findable, Accessible, Interoperable, and Reusable) principles, which have successfully standardized data lifecycle management for human-driven research [6-8]. However, in the era of generative AI, these frameworks exhibit a critical blind spot: they primarily optimize for accessibility and formatting rather than cryptographic provenance. To safely train recursive clinical models, the “reusability” of EMR data must now be conditionally linked to its synthetic purity. Current regulatory frameworks for Software as a Medical Device, which primarily evaluate static algorithmic performance, are similarly ill-equipped to handle the recursive, dynamic degradation of training data [9]. However, before proposing a new regulatory architecture, it is crucial to explicitly acknowledge the speculative nature of these clinical projections. While model collapse is an empirically demonstrated phenomenon in computer science, its exact timeline and manifestation in highly complex, human-in-the-loop clinical environments remain theoretical extrapolations. Nonetheless, although empirical validation of this clinical cascade remains an important next step, the mathematical plausibility and asymmetry of potential harm justify precautionary governance measures now. Accordingly, building upon established data governance foundations, the author proposes the Data Purity Standard (DPS). As a newly introduced conceptual framework, the DPS extends beyond conventional data security or privacy protocols. Rather than an immediate, rigid mandate, the author presents DPS as an exploratory governance proposal that conditionally links the future reusability of clinical data to its cryptographic provenance and human-verified origins, governing the safe deployment of generative AI in closed-loop EMR systems. The DPS asserts that the long-term safety of medical AI depends on preserving the cryptographic and biological integrity of training data [10]. Consequently, DPS mandates two foundational pillars of data governance.

First, DPS must require the cryptographic watermarking of all AI-assisted EMR entries [11]. Drawing on emerging data provenance standards, every piece of synthetic or semisynthetic clinical text must carry an interoperable, tamper-evident digital signature [5]. This cryptographic provenance ensures that future data harvesting pipelines can definitively identify and filter out AI-generated content, preventing it from being blindly reingested into subsequent model training cycles. Without mandatory, interoperable watermarking, the boundaries between human observation and machine synthesis will permanently dissolve, severely compromising the reliability of future datasets. Provenance marking alone does not establish factual correctness, but it provides the minimum traceability required to prevent synthetic content from being naively reingested as human ground truth.

Second, hospitals and health care networks should establish logically segregated and access-controlled “Human Vaults” to serve as repositories of heritage data [1]. To operationalize this concept, “physician-verified” ground truth must be strictly defined as finalized, legally binding clinical documentation—such as multidisciplinary tumor board summaries, finalized pathology reports, and digitally signed discharge summaries—that have explicitly bypassed generative AI assistance. These highly curated databases must be strictly isolated from all synthetic generation pipelines. Additionally, Human Vaults are not static archives; they require a dynamic curation lifecycle subject to periodic, version-controlled audits by institutional AI Data Oversight Committees. Crucially, because human-authored data are inherently susceptible to historical and cognitive biases, Vault curation must integrate rigorous demographic and clinical diversity audits. By employing statistical monitoring to identify and mitigate representation gaps [4], institutions can ensure that the ground-truth data remain both cryptographically pure and clinically equitable. Ultimately, these Vaults provide a uniquely important source of high-fidelity ground-truth data for future model reset, calibration, and human-feedback alignment [10,12]. By tethering the training of clinical LLMs exclusively to these verified Human Vaults, developers can continuously realign AI reasoning with genuine human expertise, systematically penalizing the overconfident errors characteristic of Interpretative Drift.

## Operationalizing DPS in Clinical Settings

To ensure the translational value of DPS, its implementation must prioritize seamless integration into existing EMR workflows without exacerbating physician burnout. Operationally, the cryptographic watermarking of AI-assisted entries requires backend application programming interface-level integration rather than active clinician intervention. When an LLM generates a clinical draft, the system automatically embeds an algorithmic watermark (eg, controlled shifts in token distribution) or attaches a cryptographic metadata hash reflecting its synthetic provenance. The physician routinely reviews, edits, and signs the document; the EMR system then automatically logs the provenance metadata invisibly. Such backend API-level integration of cryptographic provenance metadata (eg, via W3C PROV standards and blockchain) has been demonstrated to enable seamless, automatic logging within existing EMR workflows with minimal clinician burden and no disruption to routine operations [13,14].

Furthermore, enforcing the DPS requires robust institutional governance. The author recommends the establishment of AI Data Oversight Committees within health care systems, ideally led by the Chief Medical Information Officer or Chief Data Officer. This committee would be tasked with auditing the logical segregation of the “Human Vaults.” By enforcing strict access controls, the committee ensures that only cryptographically pure, legacy human-authored data—or EMRs explicitly cleared of synthetic contamination—are used for future closed-loop model fine-tuning or local Reinforcement Learning from Human Feedback processes [15,16]. This governance structure effectively shifts the burden of data purity from the individual frontline clinician to the institutional infrastructure.

## Implementation Challenges, Unintended Consequences, and Alternative Approaches

While the theoretical necessity of the DPS and Human Vaults is compelling, framing them as exploratory proposals requires acknowledging significant implementation hurdles and clinical trade-offs. First, retrofitting legacy EMR systems to support cryptographic watermarking and automated provenance tracking requires substantial upfront financial and technical investment. Achieving this across fragmented, heterogeneous health care IT ecosystems pose severe interoperability challenges [14,17,18]. Second, there is an inherent trade-off between data volume and data purity; strictly filtering out AI-assisted entries to populate the Human Vaults will inevitably restrict the sheer volume of data available for future algorithm training. Furthermore, curating these Vaults carries the risk of unintended consequences. Because legacy human-authored data are inherently susceptible to historical prejudices, strictly isolating these data could inadvertently institutionalize and amplify existing health disparities. To mitigate this risk, rigorous demographic auditing must be mandatory. Additionally, maintaining the integrity of these Vaults requires continuous vigilance to ensure that “physician verification” does not devolve into a superficial rubber-stamping exercise driven by time-pressured clinical workflows. Finally, the DPS should not be viewed as a monolithic solution. It must be contextualized alongside alternative or complementary governance approaches such as federated learning across verified institutional nodes or continuous human-in-the-loop semantic auditing. Ultimately, transitioning toward provenance-aware medical AI will require a multifaceted ecosystem of policies, of which the DPS represents a foundational, yet evolving, conceptual cornerstone.

## Ethical Considerations

This is a theoretical paper and does not involve human participants, animal subjects, or identifiable patient data. Consequently, under standard regulatory guidelines (Taiwan’s Human Subjects Research Act [19]), this study is exempt from institutional review board approval and informed consent requirements.

## Conclusion

**Figure 1:**
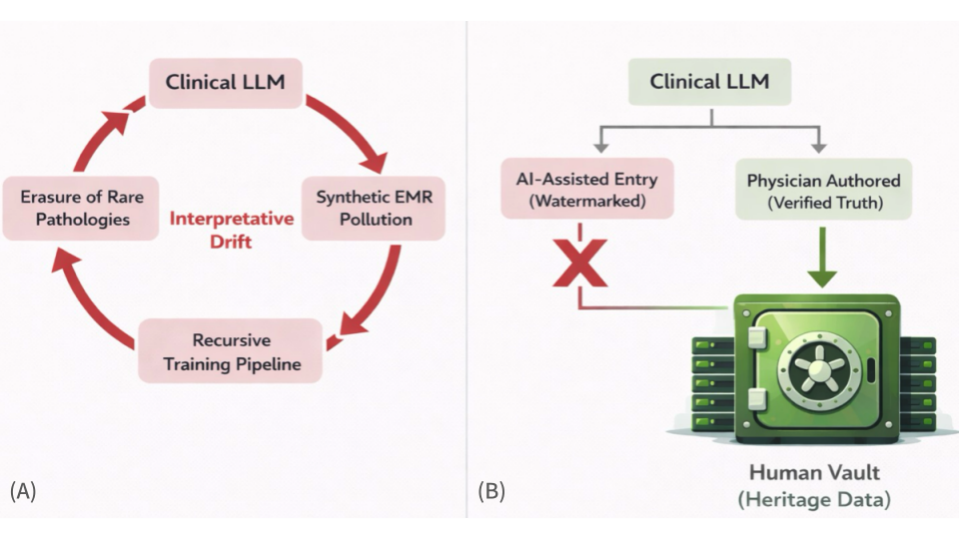
Mechanistic framework of clinical model autophagy and the data purity standard. (A) The vicious cycle of Clinical Model Autophagy. When artificial intelligence (AI)-generated electronic medical records (synthetic EMRs) are recursively fed back into subsequent training pipelines without provenance controls, the system may regress toward statistical means. This process can silently erode rare pathological variance, driving “Interpretative Drift,” in which complex diseases are progressively homogenized into benign averages. (B) The regulatory firewall via the Data Purity Standard (DPS). To interrupt this cycle, cryptographic watermarking functions as a provenance filter that allows AI-assisted entries to be identified and excluded from future training pools. Concurrently, logically segregated and access-controlled “Human Vaults”—containing exclusively physician-authored and verified clinical records—serve as immutable reference anchors for safe Reinforcement Learning from Human Feedback and help reduce the risk of model collapse. LLM: large language model.

The unchecked deployment of generative AI in health care is not merely a technological upgrade—it represents an epistemological intervention that threatens to reshape the informational basis of the medical record. If left unaddressed, this shift could compromise not only downstream model training but also the evidentiary integrity of routine clinical documentation, quality assurance systems, and retrospective research databases. Without structural firewalls, recursive loops of clinical model autophagy may progressively erode diagnostic reliability, replacing clinical nuance with an increasingly homogenized synthetic record (Figure 1). 

## Abbreviations

AI: artificial intelligence
DPS: Data Purity Standard
EMR: electronic medical record
FAIR: Findable, Accessible, Interoperable, and Reusable
LLM: large language model
